# Supplementary material for: Emergence of zero-field non-synthetic single and interchained antiferromagnetic skyrmions in thin films
Source: Nat Commun. 2022 Nov 30;13:7369. doi: 10.1038/s41467-022-35102-x (PMC9712615; doi:10.1038/s41467-022-35102-x)
Supplement: Supplementary file 1 — Supplementary Information [file 41467_2022_35102_MOESM1_ESM.pdf]

<u>Supplementary Materials</u>

Emergence of zero-field non-synthetic single and interchained antiferromagnetic skyrmions in thin films

Amal Aldarawsheh, Imara Lima Fernandes, Sascha Brinker, Moritz Sallermann, Muayad Abusaa, Stefan Blügel and Samir Lounis

a b c

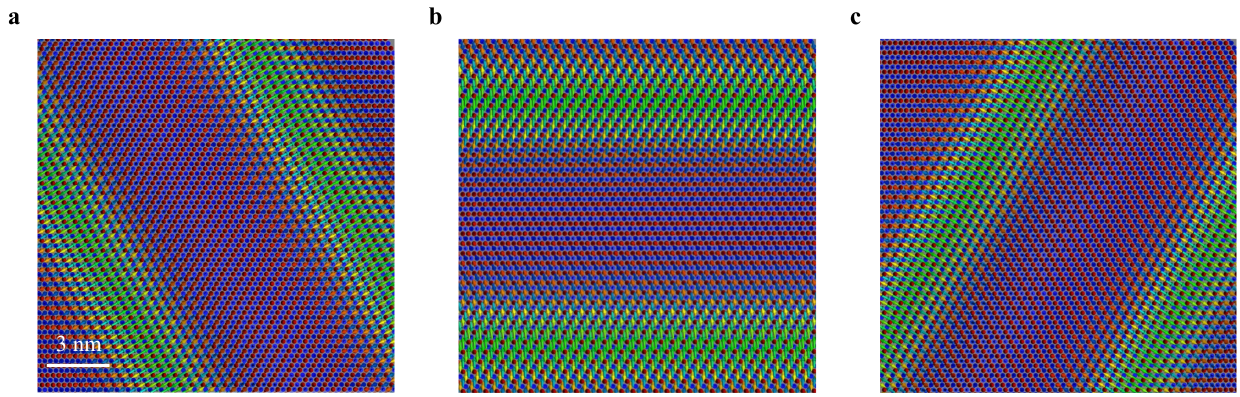

Supplementary Figure 1: Antiferromagnetic domain walls as metastable states in Cr layer deposited on PdFe/Ir(111). a, **b** and **c** Snapshots of antiferromagnetic domain walls emerging in Cr overlayer along different but equivalent orientations.

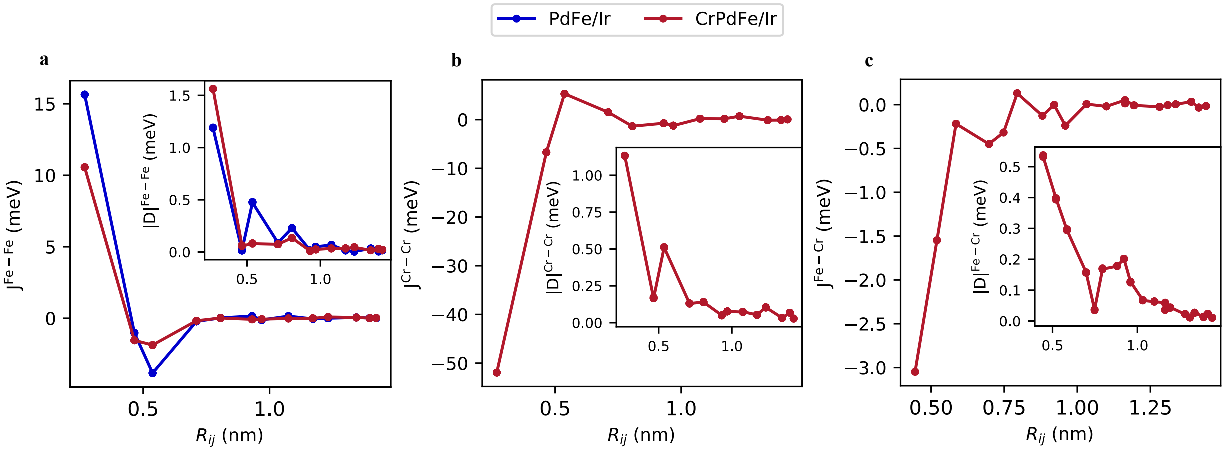

Supplementary Figure 2: Distance-dependent magnetic interactions in CrPdFe and PdFe deposited on Ir(111). **a** The Heisenberg exchange interactions as function of distance among Fe atoms ($J^{\text{Fe-Fe}}$) in PdFe/Ir(111) (blue) and in CrPdFe/Ir(111) (red), with inset indicating the corresponding magnitude of DMI. **b** The Heisenberg exchange interactions between Cr atoms ($J^{\text{Cr-Cr}}$) with DMI depicted in the inset. Similarly to **b**, the interactions between Cr and Fe atoms are shown in **c**.

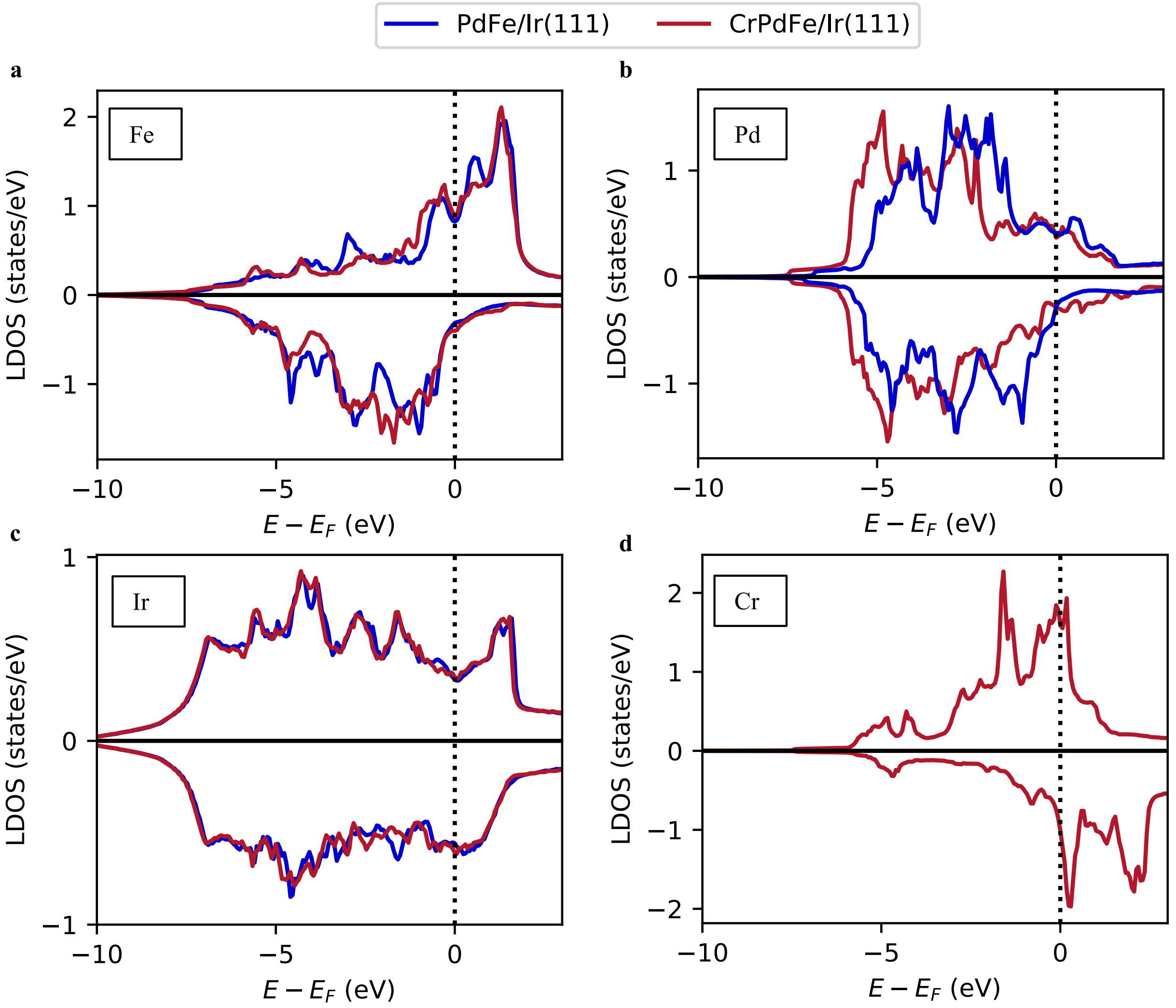

Supplementary Figure 3: Electronic structure of the CrPdFe trilayer and PdFe bilayer deposited on Ir(111) surface. Spin-resolved local density of states (LDOS) of **a** Fe, **b** Pd, **c** Ir and **d** Cr. Red and blue colors correspond to the presence or not of the Cr overlayer. The assumed magnetic state is collinear with Cr moments aligned antiferromagnetically to those of Fe.

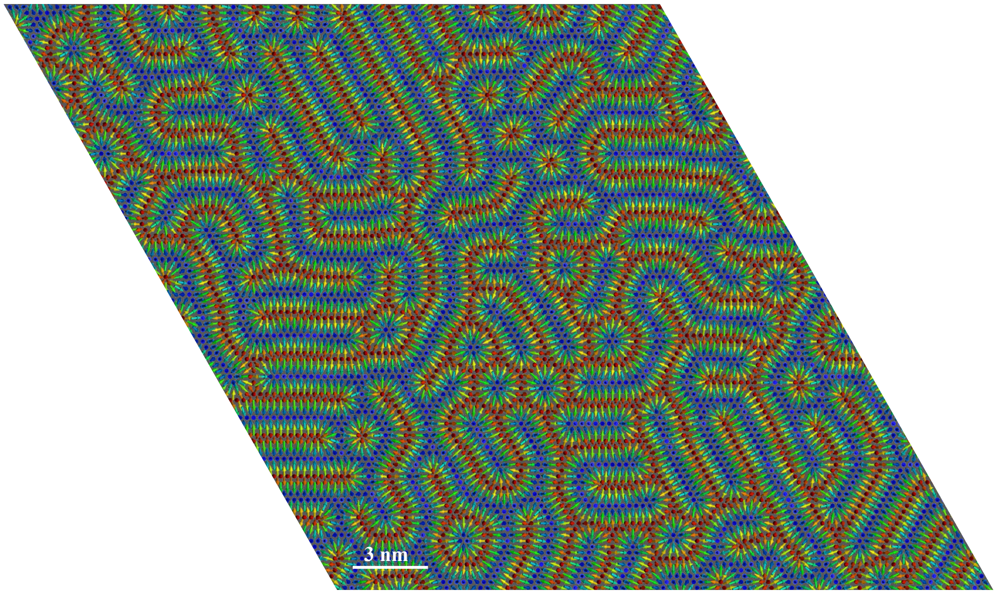

Supplementary Figure 4: Magnetic state of Fe layer when covered by the antiferromagnetic Cr layer. Ferromagnetic skyrmions emerge within the spin spirals hosted by the Fe layer without application of an external magnetic field.

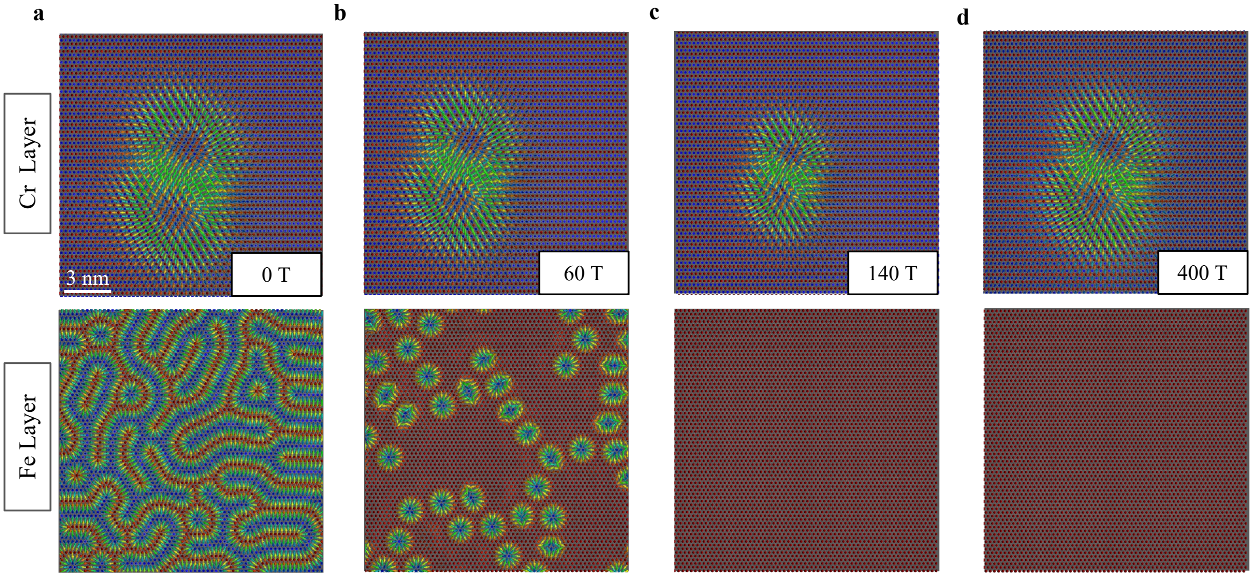

Supplementary Figure 5: Dimer of AFM skyrmion – Impact of magnetic field on the magnetic state of both Cr and Fe layers. The spin configuration in Cr (upper row) and Fe layers (lower row) for different magnetic fields applied perpendicular to the surface: 0, 60, 140 and 400T depicted at **a**, **b**, **c** and **d** respectively.

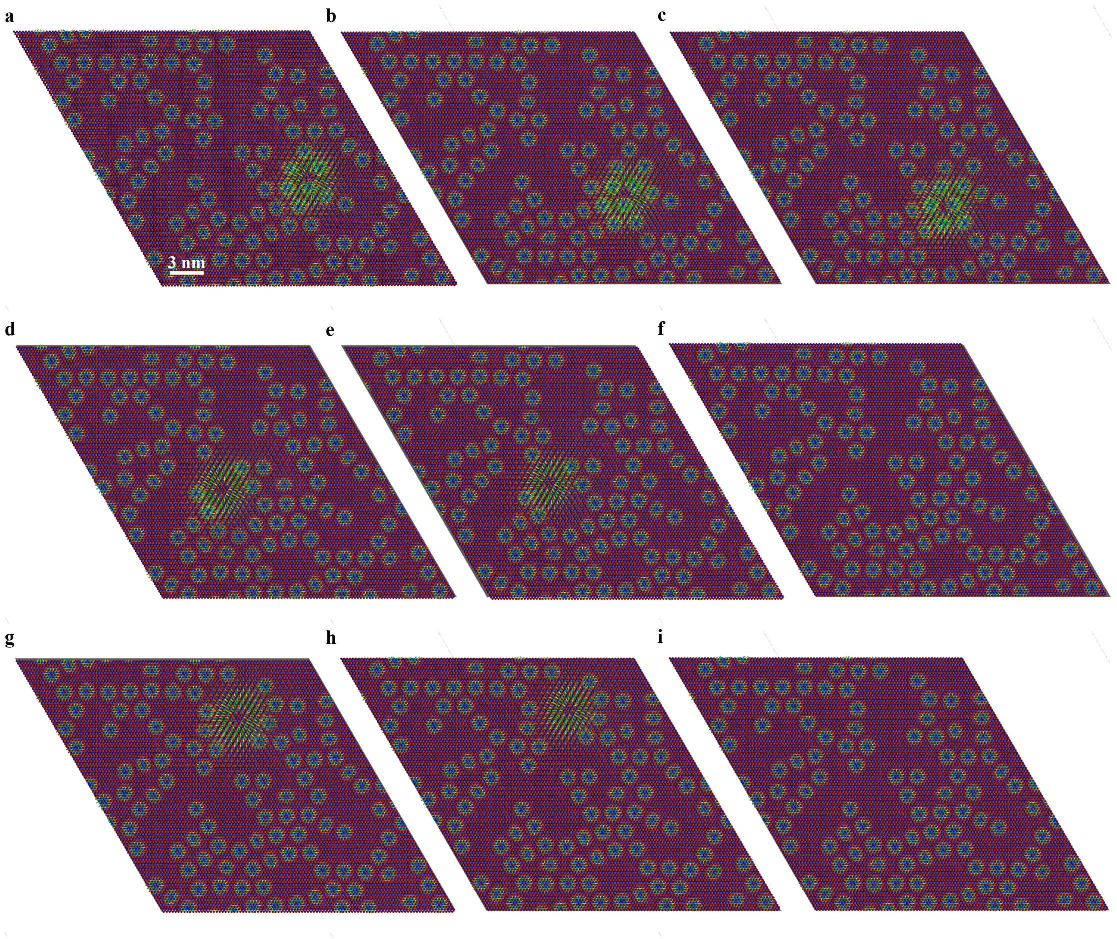

Supplementary Figure 6: Single AFM skyrmion – Stability Impacted by the magnetic inhomogeneity of Fe. Snapshots of the evolution of the single AFM skyrmion upon shifts across the lattice under a magnetic field of 70 T. The AFM skyrmion positioned directly above the ferromagnetic Fe skyrmions and antiskyrmions in **a** survives; **b** shows an intermediate state while **c** represents the final converged configuration. In **d**, the AFM skyrmion is displaced to a rather collinear region such that the skyrmion edges are rather close to the Fe skyrmions. The AFM soliton shrinks as shown in **e** before disappearing in **f**. A similar faith occurs for the AFM skyrmion shifted to a larger collinear Fe area **g** (evolution illustrated in **h** and **i**).
